# Drilling Surface Quality Analysis of Carbon Fiber-Reinforced Polymers Based on Acoustic Emission Characteristics

**DOI:** 10.3390/polym17192628

**Published:** 2025-09-28

**Authors:** Mengke Yan, Yushu Lai, Yiwei Zhang, Lin Yang, Yan Zheng, Tianlong Wen, Cunxi Pan

**Affiliations:** 1 Chongqing Engineering Research Center for Advanced Intelligent Manufacturing Technology, Chongqing Three Gorges University, Chongqing 404000, China; yanmk2023@163.com (M.Y.); 13048451325@163.com (T.W.); 19196322781@163.com (C.P.); 2 Chongqing Engineering Technology Research Center for Light Alloy and Processing, Chongqing Three Gorges University, Chongqing 404000, China; 15923400863@163.com (L.Y.); zhengyanux@163.com (Y.Z.); 3 Intelligent Manufacturing Industry Technology Research Institute, SiChuan Arts and Science University, Dazhou 635000, China; 4 School of Mechatronic Engineering, Southwest Petroleum University, Chengdu 610500, China

**Keywords:** acoustic emission, carbon fiber-reinforced polymer (CFRP), drilling, damage identification, signal processing

## Abstract

CFRP is extensively utilized in the manufacturing of aerospace equipment owing to its distinctive properties, and hole-making processing continues to be the predominant processing method for this material. However, due to the anisotropy of CFRP, in its processing process, processing damage appears easily, such as stratification, fiber tearing, burrs, etc. These damages will seriously affect the performance of CFRP components in the service process. This work employs acoustic emission (AE) and infrared thermography (IT) techniques to analyze the characteristics of AE signals and temperature signals generated during the CFRP drilling process. Fast Fourier transform (FFT) and short-time Fourier transform (STFT) are used to process the collected AE signals. And in combination with the actual damage morphology, the material removal behavior during the drilling process and the AE signal characteristics corresponding to processing defects are studied. The results show that the time-frequency graph and root mean square (RMS) curve of the AE signal can accurately distinguish the different stages of the drilling process. Through the analysis of the frequency domain characteristics of the AE signal, the specific frequency range of the damage mode of the CFRP composite material during drilling is determined. This paper aims to demonstrate the feasibility of real-time monitoring of the drilling process. By analyzing the relationship between the RMS values of acoustic emission signals and hole surface topography under different drilling parameters, it provides a new approach for the research on online monitoring of CFRP drilling damage and improvement of CFRP machining quality.

## 1. Introduction

Carbon fiber-reinforced composite materials (CFRP) are widely used in aerospace, wind power generation, medical care, sports, and other fields due to their high specific strength, high specific modulus, high temperature resistance, strong corrosion resistance, and ease of forming [1,2,3]. However, due to the low interlaminar strength and poor impact resistance of CFRP, structural connection has always been a weak link in CFRP applications [4,5]. The main connection method of carbon fiber-reinforced matrix composite structural components is mechanical connection with assembly holes, so drilling processing has also become the most common processing technology [6]. However, due to the anisotropy, poor thermal conductivity, and low interfacial bonding strength of CFRP materials, the processing mechanism is more complicated than that of ordinary metal materials. Common processing damages include delamination, burrs, tearing, and so on [7,8]. Additionally, the inhomogeneity of composite materials and the randomness of different damage mechanisms make the damage formation process a very complex phenomenon [9]. Therefore, it is essential to monitor the CFRP machining process. This enables in-depth investigation into the damage formation mechanism, thereby facilitating the enhancement of processing quality.

Researchers have conducted extensive studies in the field of damage monitoring for composite materials and employed a variety of experimental techniques to reveal the accumulation process of damage. Currently employed sensor technologies include computed tomography, microwave testing, X-ray testing, ultrasonic testing, and acoustic emission technology [10,11,12]. Since its inception, acoustic emission technology, as a new type of dynamic non-destructive testing method, has been widely applied in the research of damage detection of CFRP laminated plates due to its unique advantages of real-time performance and continuous monitoring [13].

In past few years, the application of AE technology in the damage behavior of CFRP laminates in tension, compression, and load condition was investigated by many researchers. Qiao et al. [14] carried out a 3-point bending test and established a damage pattern recognition model for carbon-fiber reinforced composites. Wavelet packet decomposition was performed on the waveforms, and the frequency bands where the main energy is concentrated and the damage pattern correlation were analyzed. Xue et al. [15] studied the indentation response of carbon fiber-reinforced composite laminates. In this work, the damage evolution behavior of carbon fiber-reinforced composite laminates during indentation experiment was studied and the location of the damaged sound source was found based on the machine learning method. Hamam et al. [16] studied induced cross-cracking-origin acoustic emission from carbon fiber/epoxy matrix composite laminates and investigated the influence of type of sensor, specimen thickness, and ply stacking sequence. The frequency content corresponding to the same damage mechanism varied significantly depending on the type of sensor and the stacking sequence. Claudia et al. [17] developed a deep learning model based on a convolutional neural network (CNN) for classifying AE waves produced by different damage patterns on carbon fiber-reinforced panel test samples. The trained CNN with Mel spectrograms of the acoustic waveforms can successfully classify the different damage modes during the failure progression of carbon-fiber reinforced polymer (CFRP) specimens. Dong et al. [18] developed a fatigue damage equation for composite laminates based on AE signals, revealing the physical mechanism of fatigue damage. The application prospect of AE technology in non-destructive health monitoring and fatigue life prediction of composite materials was proved. From the above literature review, it can be observed that most of the literature work focuses on the damage mechanism of CFRP in static service, while less research can be found on the machining damage mechanism.

Researchers have also applied acoustic emission technology to the processing of CFRP. Devin et al. [19] used acoustic emission technology to study the acoustic emission signals of drill bits with different cutting methods and geometric shapes during the drilling process, and investigated the relationship between the width of the transverse edge and the acoustic emission signals. Twardowski et al. [20] applied machine learning methods to the analysis of AE signals based on acoustic emission signals to evaluate the wear degree of milling cutters. Multiple machine learning methods were also compared, and finally the decision tree was determined to be the optimal one. Zhu et al. [21] put forward a novel integrated approach that integrates multi-characteristic and multi-signal source analysis. This method combines a backpropagation (BP) artificial neural network (ANN) model with a high-efficiency automatic system based on a sliding window algorithm, aiming to predict the tool wear condition during CFRP drilling operations. Mathiyazhagan et al. [22] employed advanced signal processing techniques and automated machine learning (AutoML) for AE signal analysis in real time, enabling accurate and efficient tool wear prediction. Nie et al. [23] studied the relationship between the characteristics of the acoustic emission signals and roughness of the CFRP inner-wall in different processing parameters and a different number of holes. Li et al. [24] used the AE method to characterize the stability of the robotic milling system. Eaton et al. [25] concluded that acoustic emission activity is associated with changes in the cutting process, and there is a correlation between tool wear and acoustic emission. Based on the above research, AE technology has shown certain practicality and prospects in CFRP processing. It mostly focuses on the relationship between the geometry of the drilling tool, the wear state of the tool, and the acoustic emission signal. There are relatively few studies on the specific correlation between the changes of AE signals and surface damage during the drilling process of CFRP materials.

Furthermore, due to the complex mechanical properties of composite materials, the drilling process of CFRP is carried out in an almost fully enclosed state. Its poor thermal conductivity easily leads to heat accumulation in the material [26]. The continuous accumulation of heat during drilling tends to soften the resin matrix, which is highly sensitive to temperature. This not only reduces the supporting and protective effect of the carbon fiber reinforcement but also weakens the physical properties of the material [27]. Studies have shown that investigating temperature changes during the machining process can guide machining operations without causing matrix damage [28]. Therefore, incorporating temperature signals during the machining process helps to gain a deeper understanding of the mechanism underlying the initiation and evolution of CFRP machining damage.

This paper mainly adopts acoustic emission technology and infrared thermography to study the characteristics of AE signals and temperature signals during the CFRP drilling process, and analyzes the variation patterns of the RMS values of AE signals and temperature signals under different machining parameters. The AE signals are processed using fast Fourier transform (FFT) and short-time Fourier transform (STFT) to determine the specific ranges of AE signals generated by matrix cracking, delamination, and fiber breakage damage during the CFRP drilling process. Finally, the CFRP surface machining quality is evaluated by combining AE signals with the topography of the hole entrance and exit. The main purpose of this study is to establish a relationship between acoustic emission signals and CFRP machining surface quality, which enables real-time monitoring and thereby facilitates the evaluation of surface quality.

## 2. Experimental Setup

The workpiece used in this experiment is a unidirectional CFRP laminate, which is made of T300 carbon fiber and LT-03A epoxy resin materials. The volume fraction of carbon fiber is 65%, and the layup direction is 0°, 90°, with a total of 36 layers. The size is 200 mm × 100 mm × 4.5 mm. The drilling experiment of CFRP was carried out on the WintecMV-45 CNC machining center (Wintec, Taiwan, China). The maximum spindle speed of the machine is 10000 rpm. During the drilling process, the temperature of the workpiece surface is recorded by a HIKMICRO H13Pro thermal imaging camera (HIKMICRO, Hangzhou, China) fixed in front of the machining center. The parameters of this instrument are as follows: the temperature measurement range is from −20 °C to 550 °C, the measurement accuracy is ±2 °C, and the frame rate is 25 Hz. Throughout the measurement, ensure that both the entire workpiece and the drill bit are within the camera’s field of view.

**Figure 1:**
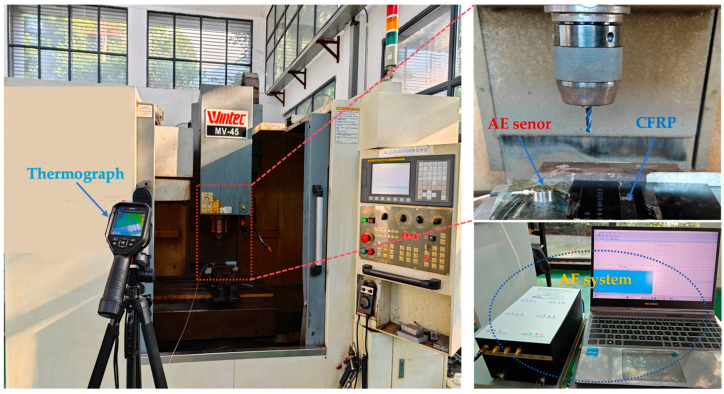
Drilling acoustic emission experiment.

**Figure 2:**
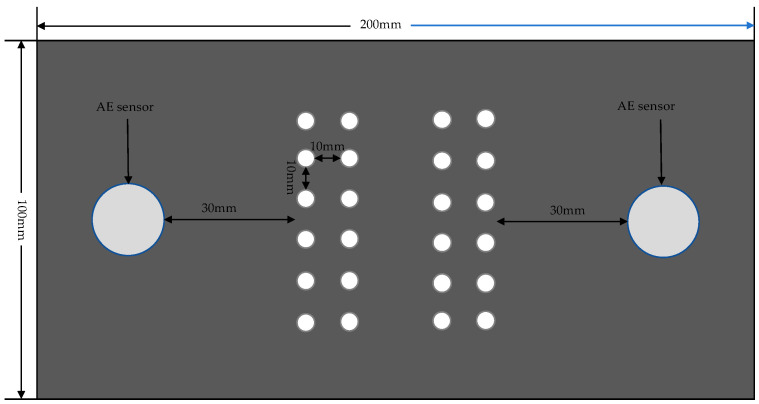
Schematic diagram of the drilling position and sensor placement.

In the drilling experiment, CFRP laminate was fixed and installed on the processing device through the fixture. The experimental tool selected is a 65-degree hard tungsten steel twist drill (KLOT, Taizhou, China) with a diameter of 5 mm and point angle of 135°. The AE measurement system includes a piezoelectric sensor (W500) (Qawrums, Guangzhou, China), preamplifier, and an AE apparatus RAEM1-6 (Qawrums, Guangzhou, China), which are responsible for the collection and recording of AE data. The operating frequency range of the W500 sensor is 100 kHz to 1000 kHz, and the AE sensor is placed 40 mm around the drilling area. The sampling rate is 2 MHz, set based on the Nyquist–Shannon sampling theorem. The gain of the preamplifier is 40 dB. Silicone grease material was applied between the AE sensor and the workpiece to reduce the attenuation of the elastic wave in the air. The sampling threshold was set at 40 dB, which was to eliminate the influence of environmental noise. The standard pencil lead breaking experiment was repeated to calibrate the AE acquisition system. The results showed that the sensor was well coupled with the workpiece surface. The AE acquisition system and experimental setup are shown in Figure 1. This paper designs a destructive experiment and a full-factor drilling experiment. For the destructive delamination experiment, a blind hole with a depth of 4.25 mm was pre-drilled in the CFRP plate first, and then the drill bit was pushed out at a rate of 5 mm/min to damage the workpiece. The parameters of the full-factor experiment are listed in Table 1, and a schematic diagram of the sensor placement and drilling position is shown in Figure 2. A total of two CFRP plates were used in this experiment: one for the destructive test and the other for the full-factor test. Thirty-two holes were drilled in the plate used for the full-factor test. All experiments were conducted under dry drilling conditions. To avoid the impact of tool wear on drilling quality, a new drill bit was used for each group of experiments, and the collected data were processed using MATLAB software (MATLAB R2022a).

## 3. Results and Discussion

### 3.1. Acoustic Emission Signal Analysis of CFRP Drilling

**Figure 3:**
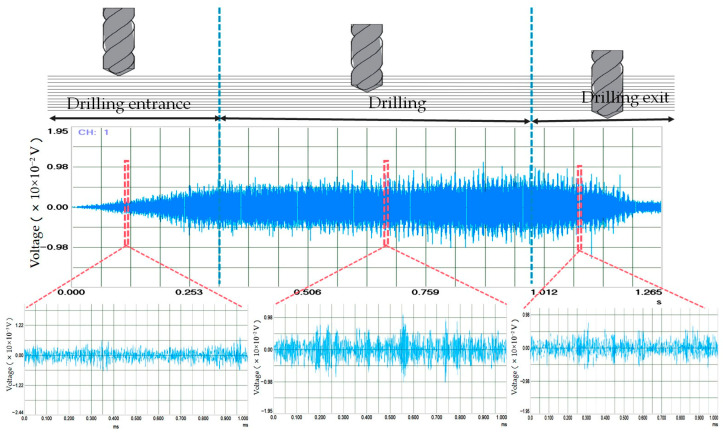
CFRP drilling acoustic emission signals and acoustic emission signals at different drilling stages.

According to References [29,30,31,32,33], fiber breakage, fiber pull-out, fiber delamination, and resin matrix fracture are the primary sources of acoustic emission during the drilling process of CFRP. By analyzing the temporal characteristics of the acoustic emission signals, the drilling process is divided into three stages: the entrance stage, stable drilling stage, and exit stage. The acoustic emission signals generated during the CFRP drilling process and the corresponding acoustic emission signals for different drilling stages are shown in Figure 3. It can be seen from Figure 3 that at the beginning of the CFRP drilling stage, the amplitude of AE signals gradually increases, and the signal fluctuates in a short period of time, which is mainly caused by the gradual increase in fiber fracture at this stage. Next, as the tool continues to go down, it enters the stable stage of CFRP drilling. It can be observed that the amplitude fluctuation of AE signals is stable and within a certain interval, but there are still abrupt signals. This is because with the drilling process, carbon fiber and resin matrix have different degrees of damage and release different acoustic emission signals. In the CFRP exit stage, the AE signal fluctuates greatly, which is because there is no support at the drilling exit, resulting in the phenomenon of fiber pull-out and matrix fracture at the exit.

**Figure 4:**
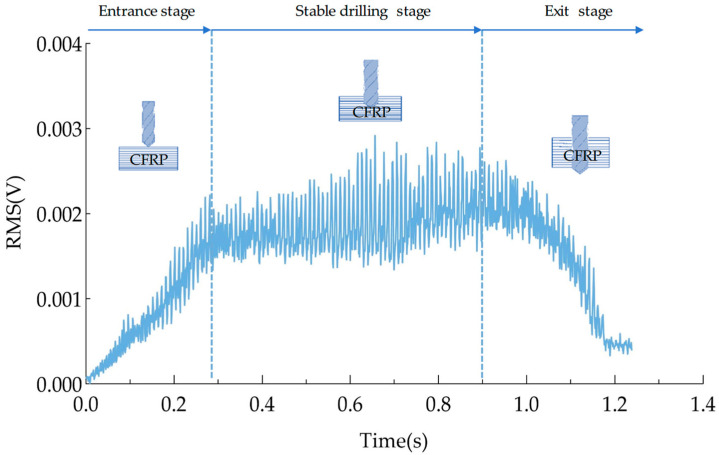
RMS when the spindle speed is 3000 rmp and the feed rate is 300 mm/min.

According to the literature of Cai et al. [34], the effective voltage value (RMS) of the AE signal can be used as an effective signal feature for machining monitoring. The effective voltage value refers to the root mean square value of the signal during the sampling time, which is related to the magnitude of acoustic emission and is mainly used for the activity evaluation of continuous acoustic emission. Figure 4 shows the RMS values of AE signals collected under machining conditions of a 3000 rpm spindle speed and 300 mm/min feed rate. Based on the machining time and changes in the RMS values, the process can be roughly divided into three stages: entrance stage, drilling stage, and exit stage. During the entrance stage, there is a significant increase in the RMS value. This is due to the continuous increase in the amount of material removed as the drilling tool contact between the drilling tool and the CFRP laminate, which leads to a rise in the signal value. The signal RMS is relatively stable during the stable drilling stage, but fluctuates within a certain range. This is due to the characteristics of CFRP materials, specifically the differences in mechanical properties between the resin base and carbon fiber materials. During the exit stage, the RMS value of the AE signal drops sharply because the amount of material removed decreases as the drilling tool breaks through the bottom surface of the CFRP plate.

### 3.2. Acoustic Emission Signal Processing

**Figure 5:**
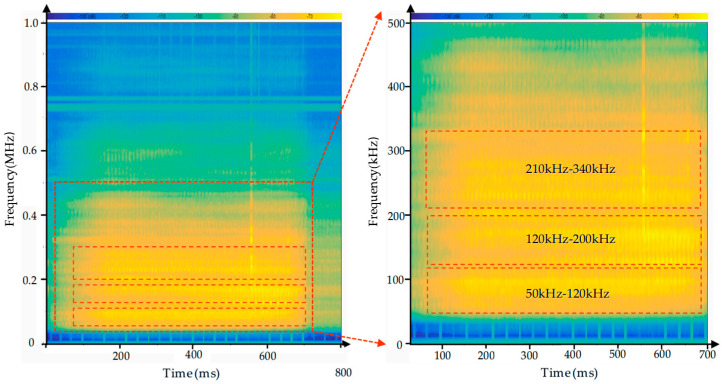
Time–frequency diagram of AE signal of drilling CFRP under the parameters of a spindle speed of 3000 rmp and feed rate of 500 mm/min.

**Figure 6:**
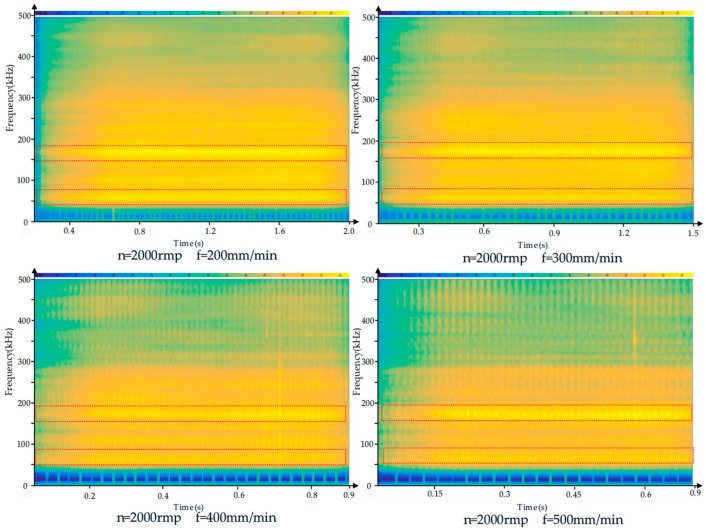
Time–frequency diagram of acoustic emission signals of CFRP drilled under the conditions of the same feed rate but different spindle speed.

**Figure 7:**
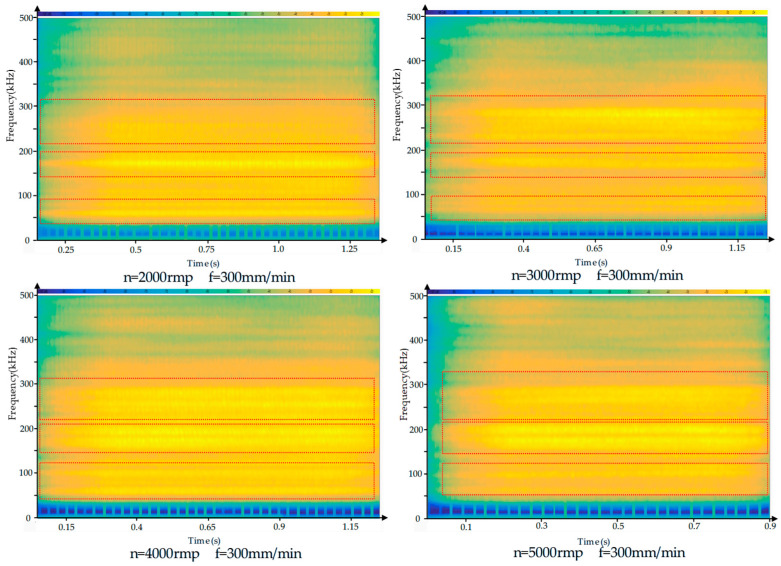
Time–frequency diagram of acoustic emission signals of CFRP drilled under the condition of the same spindle speed but different feed rate.

The acoustic emission signals generated during CFRP drilling are primarily caused by friction between the cutting tool and the resin matrix and carbon fibers in the composite material. To better understand the AE signal characteristics during CFRP drilling, short-time Fourier transform was employed for the time-frequency analysis of the AE signals. The time-frequency diagram is shown in Figure 5. As can be seen from the figure, during the drilling process, the frequency range of the acoustic emission is primarily concentrated between 0 and 500 kHz, with the most prominent frequency ranges occurring between 50 kHz and 100 kHz, 120 kHz and 200 kHz, and 210 kHz and 340 kHz. As shown in Figure 6 and Figure 7, the frequency characteristics of AE signals under different parameter conditions are presented. Due to the heterogeneity and anisotropy of CFRP composites, the removal process of CFRP materials is more complicated. After analysis, it was found that the AE signal spectra are concentrated in 50–100 kHZ, 120–200 kHZ, and 210–340 kHZ. According to the research on the frequency distribution range in the literature [35,36,37], it was found that the frequencies of matrix cracking, delamination, fiber matrix debonding, and fiber fracture are gradually increasing. We can infer that the frequency range of 50–120 kHz is related to matrix cracking, the frequency range of 120–200 kHz is related to delamination, and fiber breakage may occur within the frequency range of 210–340 kHz. Based on the above analysis, it is indicated that during the processing, the frequency characteristics of AE signals corresponding to different damage modes are less affected by processing parameters.

**Figure 8:**
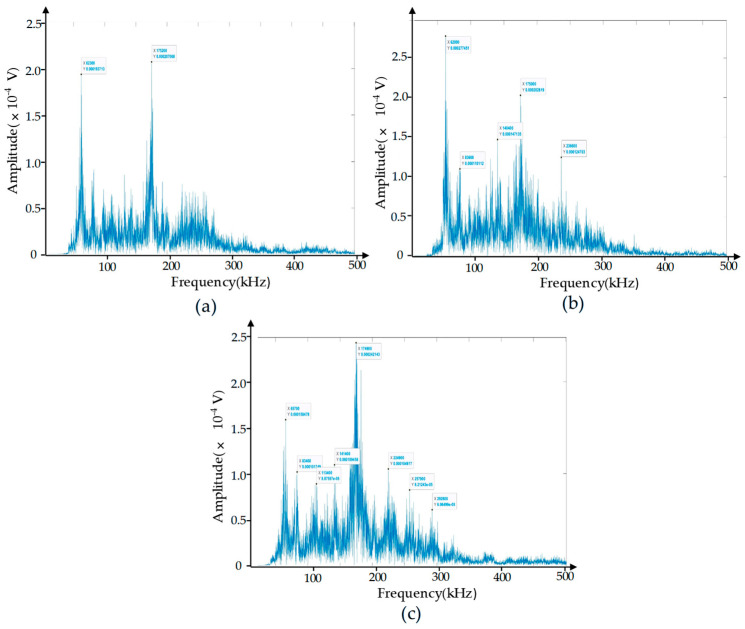
Frequency domain diagrams of AE signals. (**a**) Drilling entrance stage; (**b**) stable drilling stage; (**c**) drilling exit stage.

Since the AE signal is a transient elastic wave generated by the rapid release of energy inside the workpiece, the frequency characteristics of the AE signal corresponding to different damage modes are less affected by process parameters and other variables during the machining process. Therefore, the failure mode of CFRP can be identified by analyzing the frequency characteristics of the signal. It can be seen from Figure 7 that the highlights in the time–frequency graph are more concentrated. In order to obtain more detailed frequency domain characteristics of the acoustic emission data, the signals in the entrance stage, drilling stage, and exit stage were processed, respectively, using fast Fourier transform, and the obtained frequency distribution is shown in Figure 8. As can be seen, among these three stages, the AE signal with a frequency around 65 kHz is relatively concentrated, and we can infer that the specific frequency of matrix cracking during the drilling of CFRP composite materials is approximately 65 kHz. In the range of 120 kHz to 200 kHz, the AE signal value and frequency show a similar distribution, and the peak is concentrated around 175 kHz. However, it can be clearly seen that the signal value in the entrance stage is higher than that in the stable drilling stage and the exit stage, which may be due to the lack of support at the bottom of the CFRP workpiece during the exit stage, leading to delamination. From this, it can be inferred that the frequency of delamination is about 175 kHz. In the frequency range of 210–340 kHz, the signal in the drilling stage is not stable. Because the frequency of fiber breakage is relatively high, we can infer that fiber breakage occurred in this range.

**Figure 9:**
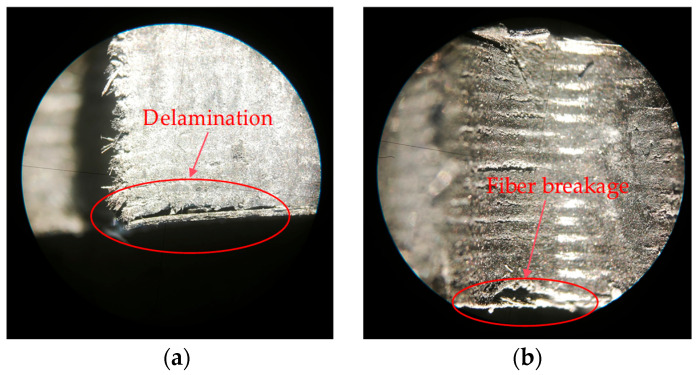
Damage morphology of destructive experiment. (**a**) Delamination damage. (**b**) Fiber breakage damage.

**Figure 10:**
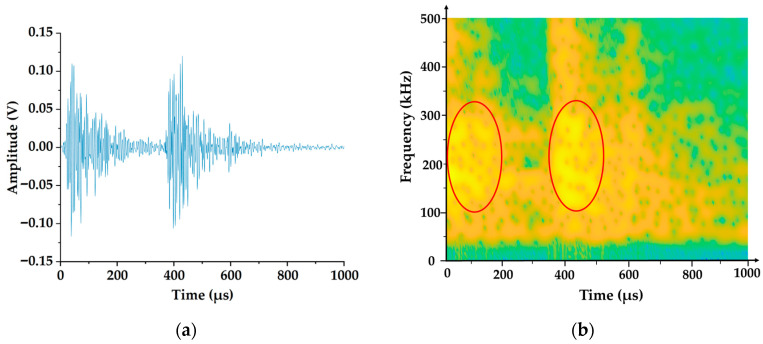
Time domain waveforms and time–frequency diagrams of acoustic emission damage signals in destructive experiments. (**a**) Time domain waveform of damage signal. (**b**) Time–frequency diagram of the damage signal.

**Figure 11:**
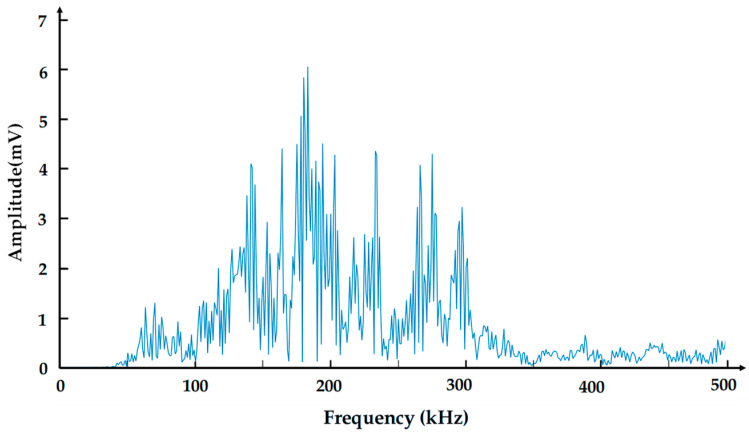
Frequency domain diagram of damage signal.

To verify the above conclusions, we designed a destructive experiment. The damage morphology of the destructive experiment is shown in Figure 9, where we can clearly observe delamination and fiber breakage. Amplify and intercept the abrupt signal in the original acoustic emission signal, and analyze the signal from the perspective of the time–frequency domain. Figure 10a shows the time domain waveform of the damage signal. We can see that after the damage occurs, the amplitude of the acoustic emission increases rapidly and then decays rapidly within a short period. This is because the damage generated an acoustic emission source, causing an abrupt change in signal amplitude. After that, the damage tended to stabilize, and the acoustic emission source gradually weakened. Figure 10b is the time–frequency diagram of the damage signal. As can be seen from the diagram, the signal intensity is relatively high in the time periods of 0–0.2 μs and 0.3–0.5 μs, which shows a high degree of consistency with the time domain waveform in Figure 10a in terms of both time and intensity. The high-energy damage frequency band is distributed between 120 kHz and 340 kHz. For a further analysis of the frequency domain characteristics of the damage signal, the spectrum diagram is presented in Figure 11, where the energy of the damage signal is concentrated in the range of 120–340 kHz. Among them, the highest energy frequencies are distributed between 120 and 200 kHz, allowing us to verify that the high-energy components in the 120–200 kHz frequency range are related to delamination damage. The high-energy components in the 210–340 kHz range are associated with fiber breakage. In addition, there is a low-frequency component distributed between 60 and 120 kHz, but its energy proportion is not high, indicating that this part is related to matrix cracking. The conclusions obtained from the verification test are consistent with those reached earlier. In the actual machining process, the size and location of damage can be determined based on the signal intensity and occurrence time in the time–frequency diagram.

### 3.3. Influence of Drilling Parameters on Acoustic Emission Signal

**Figure 12:**
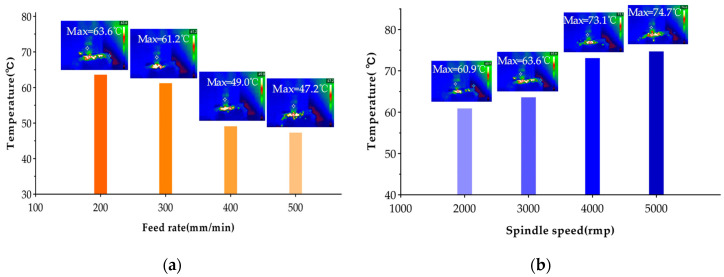
Influence of drilling parameters on temperature. (**a**) Spindle speed = 3000 rpm, feed rate = 200, 300, 400, 500 mm/min. (**b**) Spindle speed = 2000, 3000, 4000, 5000 rpm, feed rate = 200 mm/min.

During the CFRP drilling process, factors influencing the acoustic emission signal include drilling parameters, material properties, and material temperature. Firstly, to study the effect of material temperature, we need to analyze the temperature data collected by the thermal imaging camera and investigate the temperature changes on the workpiece surface as the processing parameters are altered. As shown in Figure 12, under the same spindle speed conditions, the temperature on the workpiece surface decreases as the feed rate increases. This is because as the feed rate increases, the number of times the drill bit participates in cutting CFRP decreases, resulting in reduced friction time and friction frequency between the drilling tool and CFRP material, thereby reducing the total cutting heat. Under constant feed rate conditions, the surface temperature of the workpiece increases as the spindle speed increases. Specifically, with the increase in spindle speed, the friction between the drill bit and the workpiece is more sufficient in unit time, which can generate more heat, and the smaller spindle speed is more conducive to the dissipation of heat [38]. In the drilling experiments under different parameters, there is no thermal damage to the workpiece. The highest surface temperature of the workpiece is 78.7 °C, which is much lower than the glass transition temperature of the resin matrix (180 °C). It can be judged that the material properties are not affected, and the temperature will not affect the acoustic emission signal during drilling.

**Figure 13:**
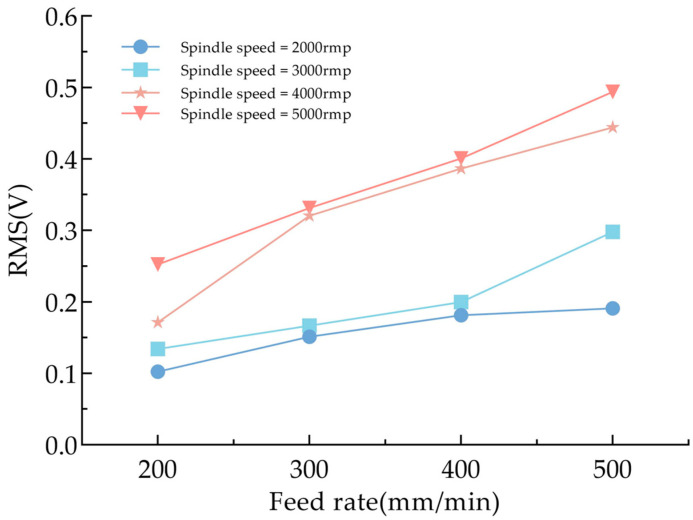
Relationship between spindle speed, feed rate, and RMS value.

We further investigated the influence of AE signals on the drilling quality of CFRP by studying the variation patterns of AE signals under different processing parameters. The average RMS value of the collected AE signals is selected as the focus of analysis, and its variations under different spindle rotational speeds and feed rates are evaluated. As illustrated in Figure 13, average RMS values are presented under conditions of spindle speeds at 2000, 3000, 4000, and 5000 rpm, and feed rates at 200, 300, 400, and 500 mm/min, respectively. The results indicate that, at a constant spindle speed, the RMS value of the AE signal increases with the increase in feed rate. This phenomenon can be attributed to the higher contact pressure between the tool and the CFRP workpiece per unit time, which leads to an increased material removal rate and intensified fiber fracture within the composite material, thereby enhancing the AE signal intensity. Similarly, when the feed rate is held constant, the RMS value also increases with rising spindle speed. This is due to the increased material removal rate and intensified friction between the drill bit and the hole wall, which results in higher drilling power and consequently stronger AE signal emissions.

### 3.4. Evaluation of Drilling Hole Damage by AE Technology

**Figure 14:**
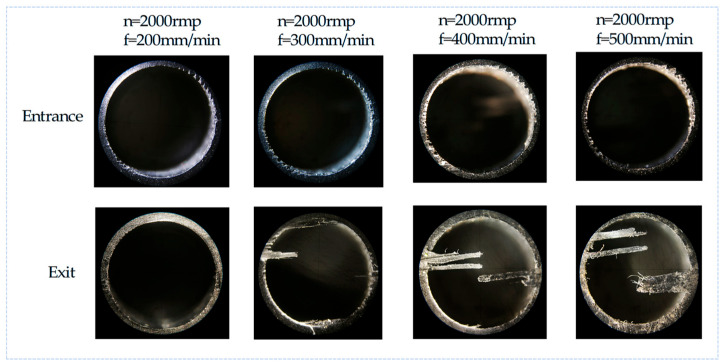
Surface topography of CFRP hole entrance and exit with the same spindle speed and increased feed rate.

**Figure 15:**
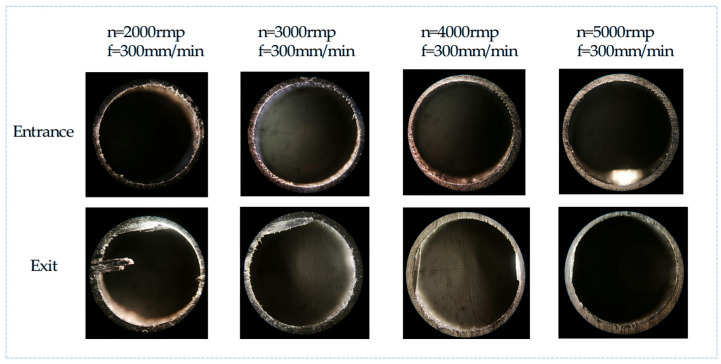
Surface topography of CFRP hole entrance and exit with the same feed rate and increased spindle speed.

**Figure 16:**
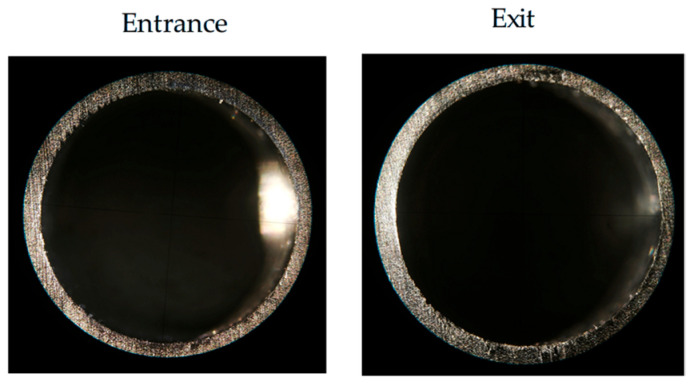
When spindle speed = 5000 rpm, feed rate = 200 mm/min; surface topography of entrance and exit.

The surface topography of the hole entrance and exit under different drilling parameters are shown in Figure 14 and Figure 15. When the tool just touches the workpiece, the carbon fiber is easily cut off due to the support provided by the workpiece, so at the entrance, almost no obvious damage is observed. However, at the exit, the material stiffness decreases with the thickness of the uncut material. When the spindle speed is constant, with an increase in feed rate, there are obvious burrs and tears at the exit of the hole. When the feed rate is constant, with an increase in spindle speed, the burr and tear damage at the exit are obviously improved. Therefore, it can be concluded that burrs and tears do not easily occur under the processing parameters of a high speed and low feed. This is because when the drill bit reaches the bottom of the material, the edge of the drill bit contacts the carbon fiber periodically. The lower feed rate increases the contact time, and the higher spindle speed increases the number of contacts, making the carbon fiber easier to remove, thereby improving the quality of the hole exit. When the spindle speed is 5000 rpm and the feed rate is 200 mm/min, the surface topography of the CFRP entrance and exit is shown in Figure 16, which can verify the conclusion.

**Figure 17:**
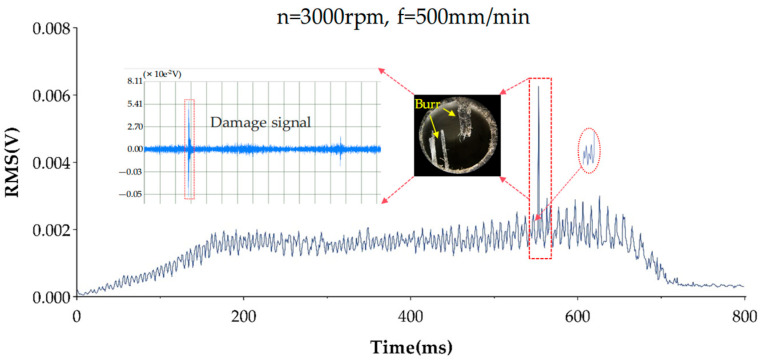
AE signal and exit topography corresponding figure.

Figure 17 illustrates the surface morphology at the hole exit along with the associated RMS plot of the AE signal, obtained under drilling conditions involving a spindle speed of 3000 rpm and a feed rate of 500 mm/min. The estimated time for the drill bit to arrive at the workpiece bottom is marked within the circular dashed boundary. During this phase, notable variations appear in the AE signal. This occurs due to the progressive exposure of the CFRP from beneath the drill tip. As the applied force surpasses the material’s maximum structural strength, fiber fractures and burr development occur, generating high-energy elastic waves. These waves are detected and recorded by the acoustic emission measurement system. Following this, the RMS level declines but remains elevated compared to the levels recorded during the steady drilling phase. This is due to the ongoing elimination of burrs, during which the tool’s interaction shifts from a cutting action to a ploughing mechanism. These observations confirm that the AE signal can serve as an effective indicator for assessing drilling damage on the workpiece surface.

## 4. Conclusions

This research employed FFT and STFT to process AE signals captured during the drilling of CFRP. It systematically analyzed the recognition and assessment of AE signals across various drilling phases and explored how the RMS values of AE signals vary under different machining conditions. The work presents an innovative approach for monitoring CFRP drilling processes through AE technology. Key findings include the following:

1. The drilling process can be categorized into three distinct stages based on variations in the RMS of AE signals. Notably, a marked rise and fall in RMS values occur during the entrance and exit stages of drilling.
2. The frequency features of AE signals associated with various damage types remain relatively stable regardless of machining parameters. These frequency domain characteristics can thus serve as indicators for identifying damage mechanisms during CFRP drilling. Specifically, collective cracking is associated with frequencies ranging from 60 to 120 kHz, delamination occurs within 120 to 200 kHz, and fiber fracture appears in the range of 210 to 340 kHz.
3. Temperature has minimal influence on AE signals during drilling. The surface temperature of the workpiece decreases with an increase in feed rate and increases with an increase in spindle speed. However, machining parameters significantly affect the RMS values. When spindle speed remains constant, the RMS value increases with higher feed rates. Similarly, with a fixed feed rate, increasing the spindle speed also leads to higher RMS values.

## Figures and Tables

**Table 1:** Experiment’s parameters.

Spindle Speed (rpm)	Feed Rate Per Tooth (mm/min)
2000/3000/4000/5000	200/300/400/500

## Data Availability

The original contributions presented in the study are included in the article; further inquiries can be directed to the corresponding authors.
